# Metabolic Differences between Subcutaneous and Visceral Adipocytes Differentiated with an Excess of Saturated and Monounsaturated Fatty Acids

**DOI:** 10.3390/genes11091092

**Published:** 2020-09-18

**Authors:** Małgorzata Małodobra-Mazur, Aneta Cierzniak, Dorota Pawełka, Krzysztof Kaliszewski, Jerzy Rudnicki, Tadeusz Dobosz

**Affiliations:** 1 Institute of Forensic Medicine, Department of Molecular Techniques, Wroclaw Medical University, Sklodowskiej-Curie 52, 50-369 Wroclaw, Poland; aneta.cierzniak@student.umed.wroc.pl (A.C.); tadeusz.dobosz@umed.wroc.pl (T.D.); 2 Department and Division of Surgical Didactics, Wroclaw Medical University, M. Curie-Skłodowskiej 66, 50-369 Wrocław, Poland; dorota.pawelka@umed.wroc.pl; 3 Department of General, Minimally Invasive and Endocrine Surgery, Wroclaw Medical University, Borowska 213, 50-556 Wroclaw, Poland; krzysztof.kaliszewski@umed.wroc.pl (K.K.); jerzy.rudnicki@umed.wroc.pl (J.R.)

**Keywords:** adipogenesis, insulin signaling, palmitic acid, oleic acid, SAT, VAT

## Abstract

Obesity is a major health problem in highly industrialized countries. High-fat diet (HFD) is one of the most common causes of obesity and obesity-related disorders. There are considerable differences between fat depots and the corresponding risks of metabolic disorders. We investigated the various effects of an excess of fatty acids (palmitic 16:0, stearic 18:0, and oleic acids 18:1n−9) on adipogenesis of subcutaneous- and visceral-derived mesenchymal stem cells (MSCs) and phenotypes of mature adipocytes. MSCs of white adipose tissue were acquired from adipose tissue biopsies obtained from subcutaneous and visceral fat depots from patients undergoing abdominal surgery. The MSCs were extracted and differentiated in vitro with the addition of fatty acids. Oleic acid stimulated adipogenesis, resulting in higher lipid content and larger adipocytes. Furthermore, oleic acid stimulated adipogenesis by increasing the expression of CCAAT enhancer binding protein β (*CEBPB*) and peroxisome proliferator activated receptor γ (*PPARG*). All of the examined fatty acids attenuated the insulin-signaling pathway and radically reduced glucose uptake following insulin stimulation. Visceral adipose tissue was shown to be more prone to generate inflammatory stages. The subcutaneous adipose tissue secreted a greater quantity of adipokines. To summarize, oleic acid showed the strongest effect on adipogenesis. Furthermore, all of the examined fatty acids attenuated insulin signaling and secretion of cytokines and adipokines.

## 1. Introduction

Obesity is characterized by a very rapid growth rate, mainly in highly industrialized countries [1]. It affects a wide array of health problems, such as metabolic disorders including insulin resistance and type 2 diabetes, cardiovascular diseases, or cancers [2]. Obesity is considered to be the result of the interaction between genetic and environmental factors; the latter mainly involve the result of increased energy intake combined with a low level of physical activity. Excessive food intake leads to adipose tissue expansion, either via enlargement of adipocytes (hypertrophy) or an increasing number of adipocytes (hyperplasia) [3].

In terms of obesity and obesity-related complications, there are considerable differences between various fat depots. Generally, visceral adipose tissue (VAT) is considered to pose a greater risk of metabolic disorders such as sclerosis, hypertension, insulin resistance, or type 2 diabetes than subcutaneous adipose tissue (SAT) [4]. What is more, VAT is more likely to become hypertrophic, while the SAT is more prone to hyperplasia [5]. In addition, the generation of inflammation due to the increased secretion of numerous cytokines is associated with VAT rather than with SAT [6].

The process of adipose tissue formation is called adipogenesis. The precursors of adipocytes are mesenchymal stem cells (MSCs), residue mostly in the stromal fraction of white adipose tissue [7,8]. MSCs possess the potential to undergo commitment and differentiation into adipocytes, chondrocytes, myocytes, and osteocytes. Numerous factors stimulate MSCs’ forward adipocyte differentiation. Generally, in vitro, IBMX, dexamethasone, and high doses of insulin are used. Following induction during the first 12 h, the step known as mitotic clonal expansion (MCE), which is several mitotic divisions, takes place. Next, after growth arrest, the early transcription factors i.e., CCAAT enhancer binding protein β (*CEBPB*) and CCAAT enhancer binding protein β (*CEBPD*) are expressed, induced directly or indirectly by the component of the differentiation medium. In turn, the early transcription factors induce the expression of late transcription factors i.e., peroxisome proliferator activated receptor γ (*PPARG*) and CCAAT enhancer binding protein α (*CEPBA*), which induce the expression of other adipogenic genes. The adipogenesis process results in fully mature adipocytes with lipid droplets accumulated in the cytoplasm [7,9]. In vivo, the physiological factors that drive adipogenesis and stimulate the differentiation of preadipocytes into mature adipocytes remain unclear.

Numerous reports have indicated the role of nutritional factors in the regulation of adipogenesis through stimulation or inhibition of this process. Indeed, nutritional factors have been shown to stimulate adipogenesis, increasing the number and size of adipocytes, especially fatty acids. A large number of publications have demonstrated the effect of fatty acids on adipogenesis, both on cell models or in vivo [10,11,12,13,14]. Most of these reports have demonstrated the adipogenic property of fatty acids through the stimulation of transcription factors such as *PPARG*. Indeed, free fatty acids were shown to act as ligands for the PPAR receptor [15].

High-fat diets (HFD) constitute one of the most common causes of obesity and obesity-related disorders. Large quantities of fatty acids may be exogenously delivered with food or synthesized endogenously [16]. Moreover, various effects can be observed, depending on the type of fatty acids. Generally, saturated fat is considered the main cause of obesity and obesity-related disorders such as insulin resistance. On the other hand, unsaturated (both monounsaturated and polyunsaturated) fatty acids have been shown to play a protective role against the induction of obesity-related metabolic disorders [17,18,19]. 

The most widely studied fatty acids are oleic acid (18:1n−9), palmitic (16:0), and stearic acids (18:0). At the same time, these are the most abundant fatty acids delivered with food. Previously it has been shown that all free fatty acids might influence both the adipogenesis and the phenotype of mature adipocytes [12,20].

Thus, we were searching for the effect of an excess of fatty acids, that is palmitic (16:0), stearic (18:0), and oleic acids (18:1n−9), on adipogenesis and the phenotype of mature adipocytes, predisposing individuals to metabolic disorders such as obesity or insulin resistance. Furthermore, we investigated the differences between SAT and VAT and the various effects of an excess of fatty acids on adipogenesis and the phenotype of mature adipocytes of two different fat depots.

## 2. Material and Methods

The study protocol was approved by the Ethics Committee of Wrocław Medical University (Ethics Committee approval No. KB-191/2016).

### 2.1. The Origin of Primary Human Mesenchymal Stem Cells

Mesenchymal stem cells (MSCs) of white adipose tissue (WAT) were acquired from adipose tissue biopsies obtained from SAT and VAT from patients undergoing abdominal surgery at the Department of General, Minimally Invasive, and Endocrine Surgery, Wrocław Medical University. All enrolled patients were informed about the proprieties of the study and gave written content before sample collection. The inclusion criteria were: (1) age of the patient (35–50 years); (2) normal BMI value (19–24 kg/m^2^); (3) fasting glucose level below 100 mg/dL; (4) absence of type 2 diabetes in close family members. An additional inclusion criterion for women was the absence of gestational diabetes in the patient’s history. Moreover, patients suffering from infectious disease, thyroid dysfunction, cancer, and alcohol-abusing patients or those with a positive history in the past were excluded.

MSCs from both VAT and SAT were extracted from 9 patients (5 women, 4 men). The mean age was 35 ± 10 years, mean BMI was 23 ± 0.5 kg/m^2^, mean fasting glucose level 88 ± 3.3 mg/dL, and mean HOMA (Homeostatic Model Assesment) 2.0 ± 0.5. The lipids profile was as follow: Triglicerydes (TG)—73 ± 31 mg/dL, Cholesterol (CHOL)—196 ± 1.5 mg/dL, High density lipoproteins (HDL)—43.5 ± 0.8 mg/dL, Low density lipoproteins (LDL)—138 ± 8.4 mg/dL.

Immediately following dissection, the samples were placed in PBS with protease inhibitors and transported to the laboratory, where the MSCs were extracted according to the protocol described previously [20]. Briefly, visible blood vessels were removed; tissue was separated with scissors and treated with 1 mg of collagenase (Sigma-Aldrich, St. Louis, MO, USA) with the addition of 10 mg of BSA (Bovine Serum Albumin) (Sigma-Aldrich, St. Louis, MO, USA) per 1 mL of DMEM (Dulbecco’s Modified Eagle Medium)) at 37 °C for approximately 1 h, until complete digestion. Next, the collagenase was inactivated by adding DMEM equaling five volumes of the initial volume of the digestion mixtures. Samples were left at RT (Room temperature) for 10 min to allow the adipocytes to float to the surface. The adipocytes were removed, and the rest of the sample was centrifuged at 4 °C for 5 min at 2000 rpm. The DMEM was discarded and the pellet was washed with PBS, then centrifuged at 4 °C for 5 min at 2000 rpm. The cells were suspended in DMEM/F12 (50:50, Corning, New York, NY, USA) with 10% FCS (Fetal Bovine Serum) (Corning, New York, NY, USA) and antibiotics (50 U/mL of penicillin and 50 µg/mL of streptomycin, Corning, New York, NY, USA) and plated on a 7-mL culture plate. Cells were cultured in DMEM/F12 (50:50) with 10% FCS and antibiotics until confluence. The culture medium was replaced every second day.

Before the experiments, the viability test using an MTT (3-(4,5-dimethylthiazol-2-yl)-2,5-diphenyltetrazolium bromide, a tetrazole, Sigma-Aldrich, St.Louis, MO, USA) assay was performed to assess the highest concentration of fatty acids (16:0, 18:0, 18:1n−9) not affecting the viability of the cells. The wide range of concentration of appropriate fatty acids was tested (0.1–1 mM). For controls, an equal value of bovine serum albumin (BSA) was added. Briefly, cells were incubated for 24 h and 48 h with fatty acids or BSA; next 5 mg/mL of MTT solution was added, and the samples were further incubated for an additional 3, 5 h at 37 °C. After incubation, purple formazone was dissolved in DMSO (Corning) and the absorbance was measured at 590 nm with a reference filter of 620 nm. The viability test was repeated three times. Based on obtained results the concentration 0.5 mM was chosen as the highest concentration not affecting cell viability.

### 2.2. Adipogenesis of Human MSC

A total of 24 h before adipogenesis induction, 0.5 mM fatty acids were added (palmitic 16:0, stearic 18:0 and oleic 18:1n−9 acids). For controls, an appropriate amount of BSA fatty acids-free was added.

Adipogenesis was induced by a differentiation media containing DMEM/F12 (50:50), 10% FCS, penicillin (50 U/mL), streptomycin (50 µg/mL), IBMX (115 µg/mL), dexamethasone (390 ng/mL), insulin (10 µg/mL), pioglitazone (0.1 µg/mL), human transferrin (10 µg/mL), and 0.5 mM of particular fatty acids (palmitic 16:0, stearic 18:0 and oleic acid 18:1n−9) and BSA for controls. All reagents for adipogenesis, including fatty acids were purchased from Sigma-Aldrich. Cells were kept in the differentiation medium for 3 days. Next, the medium was replaced with a medium containing DMEM/F12 (50:50), 10% FCS, penicillin (50 U/mL), streptomycin (50 µg/mL), insulin (10 µg/mL), pioglitazone (0.1 µg/mL), human transferrin (10 µg/mL), and 0.5 mM of particular fatty acids and incubated for 4 more days. For an additional 3 days, cells were incubated in DMEM/F12 (50:50), 10% FCS, the antibiotic mixture, and 0.5 mM of a particular fatty acid until final maturation. To the control cells, albumin of appropriate concentration was added.

The progress of adipogenesis was monitored at five time points: day 0 (D0)—the time of adipogenesis induction, day 0.5 (D0.5) 12 h after adipogenesis induction (the MCE stage), day 4 after adipogenesis induction (D4), day 7 (D7), and day 10 (D10)—the stage of fully mature adipocytes. At each selected time point, the cells were harvested and subjected to specific analysis.

### 2.3. Fatty Acids Preparation [25 mM]

The fatty acids were prepared as described previously [12]. Briefly, fatty acids were dissolved in 0.1 M NaOH (Sigma-Aldrich) and incubated for 30 min at 70 °C. Next, 10% of fatty acids-free bovine serum albumin (BSA, Sigma-Aldrich) was added and incubated further for 1 h at 55 °C with constant stirring. Finally, the fatty acids were filtered through a 0.2-µm syringe filter and stored at −20 °C.

### 2.4. Genetic Material Extraction

DNA from collected cells was extracted using the phenol-chloroform method. Harvested cells were washed twice with sterile PBS. The cell pellet was suspended in adipocytes lysis buffer (Tris-HCl (pH 7.5), TEN (Tris, EDTA, NaCl), 10% SDS (Sigma-Aldrich, St. Louis, MO, USA) with the addition of proteinase K (Sigma Aldrich, St. Louis, MO, USA). Next, the phenol:chloroform:isoamyl alcohol (25/24/1, v/v/v, BioShop, Burlington, Canada) was added followed by vigorous vortexing for 20 s. DNA was precipitated with 96% ethanol (StanLab, Lublin, Poland), washed with 70% ethanol, and dried in air. DNA was resuspended in DEPC (Diethyl pyrocarbonate)-treated water.

Total cellular RNA was extracted using Tri-Reagent (Sigma-Aldrich, St. Louis, MO, USA). Following the cell lysis, chloroform was added and, after centrifugation at 4 °C, RNA was precipitated with isopropanol (Sigma-Aldrich, St. Louis, MO, USA). The RNA pellet was washed with 70% ethanol (StanLab, Lublin, Poland), dried in air, and finally dissolved in DEPC-treated water.

### 2.5. Gene Expression

The cDNA was synthesized with the use of a High-Capacity cDNA Reverse Transcription Kit (ThermoFisher Scientific, Waltham, MA, USA), using 1000 ng of total RNA. Gene expression was measured using the Fast SYBR Green Master Mix (ThermoFisher Scientific, Waltham, MA, USA). Primers were designed manually to flank two adjacent exons of mRNA. The primers sequences are located in Table 1. The primers specificity was investigated using Primer-BLAST (NCBI), further checked based on the denaturation curve. The possible secondary structures of designed primers were analyzed using OligoAnalyzer (IDT, Inc., Coralville, IA, USA). The primers efficiency was analyzed using the standard curve method. Gene expression was calculated using the delta–delta Ct (ΔΔCt) model, normalizing to the reference gene (*β-actin* gene).

### 2.6. Glucose Uptake

Measurement of glucose uptake was carried out on the mature adipocytes (on day 10 of differentiation) using a Glucose Uptake-Glo Assay (Promega, Madison, WI, USA) according to protocol. Before the experiment, the cells were starved in a serum-free medium overnight. On the day of the experiment, the medium containing glucose was removed, and cells were washed twice with sterile PBS to wash out any remaining glucose. Next, cells were stimulated with 1 µM insulin for 20 min, followed by incubation with 1 mM of 2-deoxyglucose (2DG) for an additional 20 min. Finally, the reaction mixture was added, and the signal was detected by luminescence readers.

### 2.7. Adipokine Levels

Levels of adipokines (leptin, adiponectin, and Interleukin-6 (IL-6)) were investigated on D10 of differentiation in a medium collected from mature adipocytes using commercial kits: Human IL-6 PicoKine™ ELISA Kit (Boster, San Mateo, CA, USA), Human Leptin PicoKine™ ELISA Kit (Boster, San Mateo, CA, USA), and a Human ADIPOQ/Adiponectin ELISA Kit (Sigma-Aldrich, St. Louis, USA).

### 2.8. Assessment of the Accumulated Lipids

The amount of accumulated lipids was measured at the end of adipogenesis (D10 of adipogenesis induction) using Oil Red-O (Sigma-Aldrich, St. Louis, MO, USA). The cells were fixed with 4% paraformaldehyde (PFA, Sigma-Aldrich, St. Louis, MO, USA) for 10 min at RT. The PFA was removed, cells were washed with water, and further incubated with 60% isopropanol (Sigma-Aldrich, St. Louis, MO, USA) for an additional 5 min. The staining solution (Oil Red-O Stain, Sigma-Aldrich, St. Louis, MO, USA) was added and incubated at RT for 30 min. Next, the staining solution was discarded, cells were washed several times with water. The accumulated Oil Red-O was extracted with 100% isopropanol. The absorbance was measured at 492 nm.

### 2.9. Assessment of the Size of Adipocytes

The size of adipocytes was determined using Olympus IX81 using CellSens Dimension software.

### 2.10. Statistical Analysis

Statistical analysis was done with the use of Statistica 13.1 (StatSoft, Tulsa, OK, USA). The numerical values between studied groups were compared using one-way and/or multifactor ANOVA/MANOVA and the post-hoc test (the Fisher’s Least Significant Difference–LSD test). The differences between SAT and VAT were estimated using a *t*-test. Statistical significance was set at *p* < 0.05

## 3. Results

### 3.1. Adipogenesis of Stem Cells with the Addition of Fatty Acids

#### 3.1.1. Morphological Changes and Lipid Accumulation

**Figure 1:**
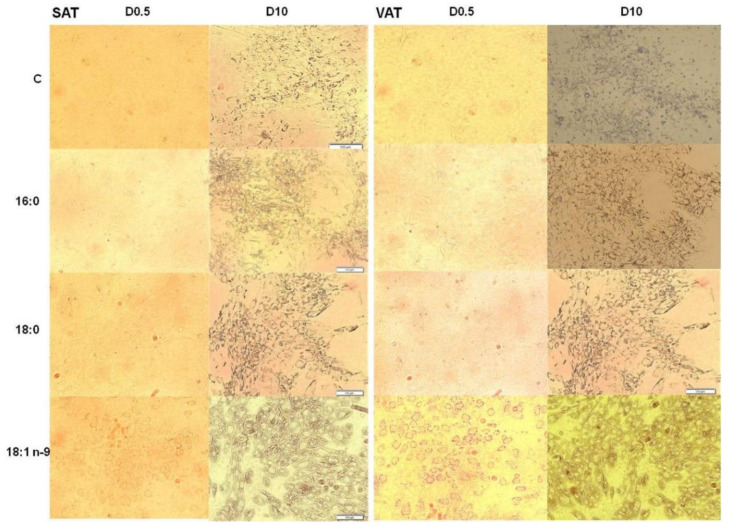
Mesenchymal stem cells (MSCs) collected from subcutaneous adipose tissue (SAT) and visceral adipose tissue (VAT) during adipogenesis. Subcutaneous (left panel), visceral (right panel): D0.5 after differentiation induction and D10 after differentiation induction—fully mature adipocytes differentiated with the excess of palmitic acid (16:0), stearic acid (18:0), oleic acid (18:1n−9), and control cells (C).

We observed that the MSCs differentiated with an excess of oleic acid (18:1n−9) faster than either control cells or two other experimental cells (differentiated with palmitic (16:0) and stearic (18:0) acids). On day 0.5 (D0.5) following the addition of differentiation medium, cells cultured with oleic acid began to change their structure into round cells with lipid droplets located around the cytoplasm (Figure 1). In control cells and cells cultured with saturated fat, the described changes began to appear around D4.

**Figure 2:**
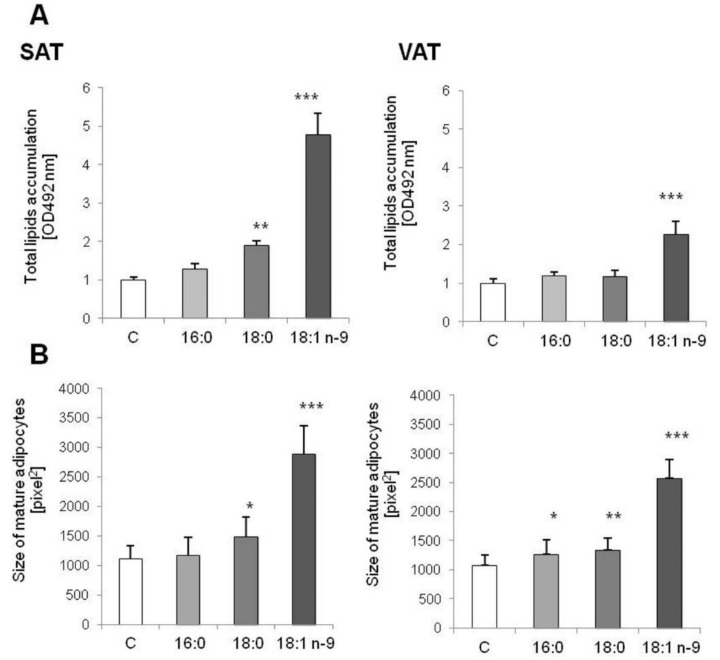
Total lipid content accumulated during adipogenesis in control and experimental cells. Palmitic acid (16:0), stearic acid (18:0), oleic acid (18:1n−9), control cells (C) (**A**). The size of mature adipocytes treated with palmitic acid (16:0), stearic acid (18:0), oleic acid (18:1n−9) and control cells (C) (**B**); * *p* < 0.05, ** *p* < 0.001, *** *p* < 0.0001.

At the end of adipogenesis, total lipid accumulation and the size of adipocytes were analyzed. We observed that oleic acid stimulated total lipid accumulation both in SAT and VAT (*p* = 0.0000); however, total lipid content was two times higher in cells obtained from SAT than from VAT (*p* = 0.0000). We also observed statistically increased lipid content in adipocytes collected from SAT treated with stearic acid (*p* = 0.0002), but not in VAT (*p* = 0.0610, Figure 2A). Total lipid accumulation corresponded to the size of adipocytes. Adipocytes differentiated with an excess of oleic acid were significantly larger than control cells (*p* = 0.0000, both SAT and VAT: Figure 2B). Similarly, adipocytes differentiated with an excess of stearic acids were larger than controls (*p* = 0.0031 for SAT and *p* = 0.0003 for VAT). The palmitic acid influenced the size of adipocytes collected from VAT (*p* = 0.0063, Figure 2B).

#### 3.1.2. Transcription Factors Regulating Adipogenesis

**Figure 3:**
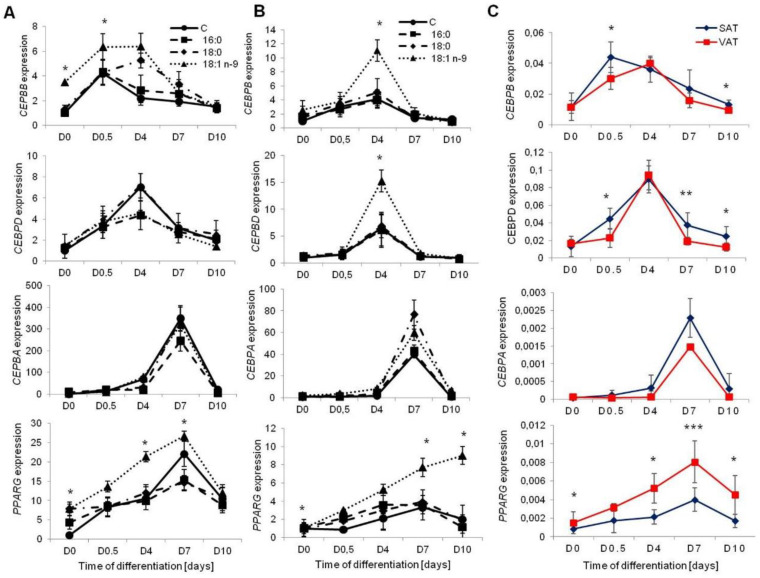
The expression of adipogenic transcription factors. Early transcription factors (CCAAT enhancer binding protein β (*CEBPB*), CCAAT enhancer binding protein β (*CEBPD*)) and late transcription factors (CCAAT enhancer binding protein α(*CEBPA*), peroxisome proliferator activated receptor γ (*PPARG*)) in SAT (**A**), VAT (**B**) in adipocytes treated with palmitic acid (16:0), stearic acid (18:0), oleic acid (18:1n−9) and control cells during adipogenesis, normalization was done to the day 0 (D0). Expression of transcription factors between SAT and VAT (**C**); * *p* < 0.05, ** *p* < 0.001, *** *p* < 0.0001.

To assess the progress of adipogenesis, we investigated the expression of the main adipogenic transcription factors: two early (*CEBPB* and *CEBPD*) and two late (*CEBPA* and *PPARG*) transcription factors. The expression of *CEBPB* was higher in cells from SAT differentiated with an abundant concentration of oleic acid at D0 and D0.5 (*p* = 0.0016 and *p* = 0.0013, respectively) compared to control cells. Similarly, oleic acid stimulated the expression of *CEBPB* in VAT-derived adipocytes (D4 *p* = 0.0135: Figure 3).

*PPARG* was overexpressed in VAT-derived MSCs differentiated with the addition of oleic acid through the entire adipogenesis process; however, a significant increase was noted at D0 (*p* = 0.0281), D7 (*p* = 0.0194), and D10 (*p* = 0.0335) (Figure 3B). Similarly, in SAT-derived cells, the expression of *PPARG* was considerably greater throughout the adipogenesis process, with significant differences at D0 (*p* = 0.0432), D4 (*p* = 0.0325), and D7 (*p* = 0.0435) of differentiation (Figure 3A). We also observed a considerable increase in the expression of *PPARG* in preadipocytes treated with stearic acid; however, the increase was significant only at D0 of differentiation (*p* = 0.0354).

We also investigated potential differences in the expression rate of transcription factors during the adipogenesis of the MSCs collected from visceral and subcutaneous fat. The early transcription factors (*CEBPB* and *CEBPD)* were expressed at a higher rate in cells collected from SAT. Among the late transcription factors, *PPARG* was expressed at a higher level in VAT-derived adipocytes throughout the process of adipogenesis (D0, *p* = 0.0013; D0.5, *p* = 0.0571; D4, *p* = 0.0437; D7, *p* = 0.0001; D10, *p* = 0.0154, Figure 3C).

### 3.2. The Phenotype of Mature Adipocytes

The phenotype of adipocytes differentiated with the excess of fatty acids was investigated at the end of adipogenesis (on mature adipocytes). The metabolic markers of insulin signaling, the secretion rate of cytokines and adipokines, and the lipid metabolism were measured. Finally, the metabolic differences between cells from SAT and VAT were assessed. In the last-named case, comparisons were made between control cells only, differentiated at standard conditions without the excess of fatty acids.

#### 3.2.1. Insulin Signaling Pathway

To assess insulin sensitivity, the expression of main genes belonging to the insulin-signaling pathway was measured.

**Figure 4:**
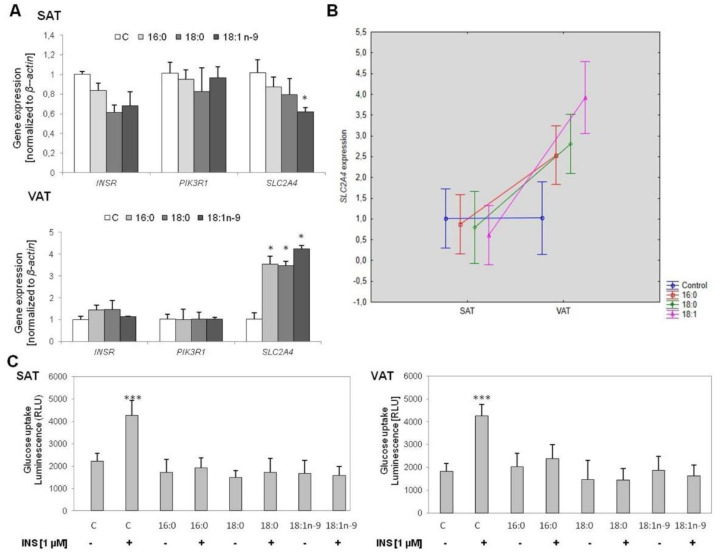
The expression of insulin pathway genes in SAT and VAT adipocytes. Comparison of gene expression (**A**), the various effect of the examined fatty acid excess on solute carrier family 2 member 4 (*SLC2A4*) expression depending on fat depot (**B**) in experimental cells (treated with palmitic acid (16:0), stearic acid (18:0), and oleic acid (18:1n−9)) and control cells. Glucose uptake test after insulin stimulation (**C**) in experimental cells (treated with palmitic acid (16:0), stearic acid (18:0), and oleic acid (18:1n−9)) and control cells. (C): * *p* < 0.05, *** *p* < 0.0001.

In adipocytes collected from SAT, no significant changes were noted in the expression of insulin receptor (*INSR*) and phosphoinositide-3-kinase regulatory subunit 1 *(PIK3R1*) genes within the investigated cells; however, the expression of solute carrier family 2 member 4 (*SLC2A4*) was downregulated in adipocytes treated with oleic acid (*p* = 0.0492) (Figure 4A).

In VAT-derived adipocytes, no significant changes were noted in the expression of *INSR* and *PIK3R1* genes within the investigated cells. On the other hand, *SLC2A4* was considerably overexpressed in all cells treated with fatty acids (*p* = 0.0340, *p* = 0.0352, and *p* = 0.0360 for palmitic (16:0), stearic (18:0), and oleic acids (18:1n−9), respectively: Figure 4A).

The expression of insulin signaling genes was similar between SAT and VAT adipocytes. However, we observed different effects of the increased concentration of fatty acids on the expression of *SLC2A4* in VAT-derived cells, which was increased by investigated fatty acids (*p* = 0.0061, *p* = 0.0020, and *p* < 0.0000 for palmitic (16:0), stearic (18:0), and oleic acids (18:1n−9), respectively: Figure 4B).

Next, we measured insulin-stimulated glucose uptake in both types of mature adipocytes. In both types of control cells, we showed an increase in glucose uptake rate following insulin stimulation (*p* < 0.0000 for both SAT and VAT); on the other hand, we detected no increase in glucose uptake following insulin stimulation in cells treated with fatty acids throughout the adipogenesis (Figure 4C). The rate of glucose uptake in the basal stage was the same as that following insulin stimulation. A similar effect was observed regardless of the type of fatty acids.

#### 3.2.2. Cytokine and Adipokine Expression and Secretion

Adipose tissue, apart from its basic role of energy storage, plays an enormous role as an endocrine organ. Accordingly, we were interested in how the excess of fatty acids influence adipokines secretion and inflammatory-stage induction.

We observed no effect of free fatty acids on inflammatory induction. Rates of *IL-6* and *IL-10* expression and IL-6 secretion were unchanged following the addition of fatty acids, which may suggest that free fatty acids do not induce inflammation during adipogenesis, although they may do so later in the life of adipocytes.

**Figure 5:**
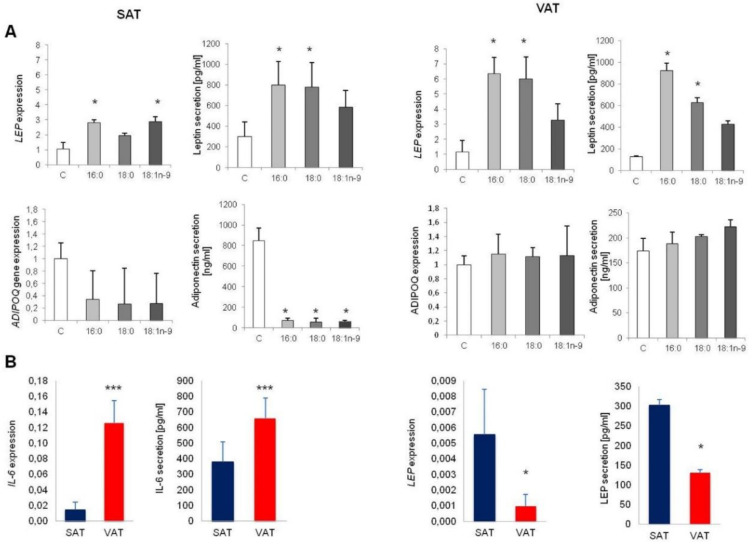
The adipokines expression and secretion in SAT and VAT adipocytes. Comparison of gene expression of control adipocytes and experimental cells (treated with palmitic acid (16:0), stearic acid (18:0), and oleic acid (18:1n−9)) obtained from SAT and VAT (**A**). Differences between Interleukin 6 (IL-6) and Leptin (LEP) expression and secretion between adipocytes obtained from VAT and SAT (**B**); * *p* < 0.05, *** *p* < 0.0001.

However, adipokines showed divergences in both expression and secretion rates under the influence of fatty acids. Both the expression and secretion of leptin were stimulated by fatty acids in adipocytes collected from SAT. The expression of the Leptin (*LEP*) gene was three times higher in adipocytes differentiated with palmitic acid, 16:0 (*p* = 0.0202), and oleic acid, 18:1n−9 (*p* = 0.0247) and twice as high in adipocytes cultured with an excess of stearic acid, 18:0 (*p* = 0.0505) compared to controls. Similarly, leptin secretion was induced, particularly by palmitic (*p* = 0.0271) and stearic (*p* = 0.0298) acids (Figure 5A). Similarly, fatty acids increased the expression of the *LEP* gene in adipocytes collected from VAT. In particular, palmitic and stearic acids increased the expression of the *LEP* gene approximately six-fold compared to controls (*p* = 0.0147 and *p* = 0.0202, respectively). Oleate increased the expression of the *LEP* gene approximately three-fold compared to controls and was near-significant (*p* = 0.0506). The secretion of leptin was increased in adipocytes differentiated with the addition of palmitate (*p* = 0.0298) and stearate (*p* = 0.0371).

The adiponectin level decreased in cells differentiated with an excess of fatty acids collected from the SAT, in terms of both expression and secretion levels. The drop in expression was similar for all investigated fatty acids; however, due to a very high standard deviation, it did not reach the level of significance (*p* = 0.1012, *p* = 0.1287, and *p* = 0.1641 for palmitic (16:0), stearic (18:0), and oleic acids (18:1n−9), respectively). The secretion of adiponectin was considerably reduced in cells differentiated with an excess of all of the investigated fatty acids (*p* = 0.0291, *p* = 0.0188, and *p* = 0.0189 for palmitic (16:0), stearic (18:0), and oleic acids (18:1n−9), respectively). VAT-derived cells showed no changes in either the expression of the *ADIPOQ* gene or the amount of secreted adiponectin in terms of fatty acids (Figure 5A).

Comparing two fat depots, we observed a significant increase in *IL-6* gene expression (*p* < 0.0000) and IL-6 secretion (*p* < 0.0000) in VAT-derived adipocytes (Figure 5B) compared to cells obtained from SAT. On the other hand, *LEP* gene expression and Leptin secretion were considerably increased in SAT-derived adipocytes (*p* = 0.0394 and 0.0152, respectively: Figure 5B).

#### 3.2.3. Lipid Metabolism

**Figure 6:**
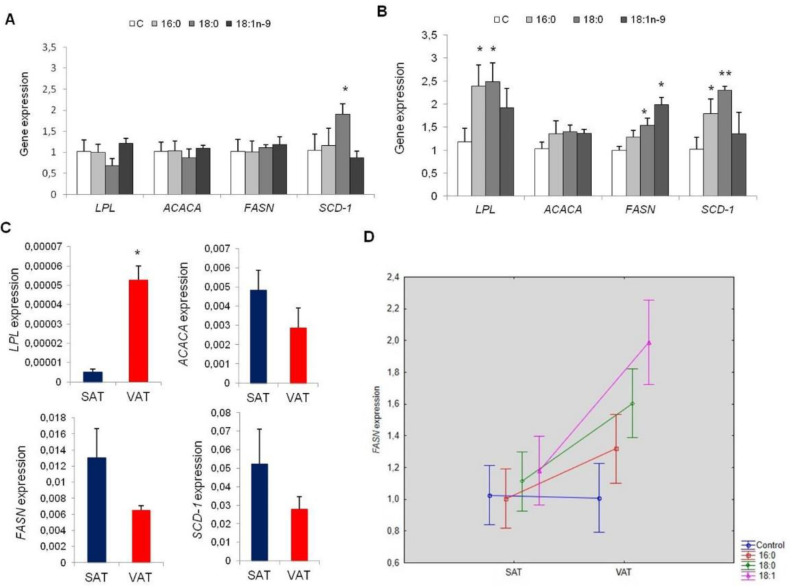
The expression of lipid metabolism genes in SAT and VAT adipocytes. Comparison of gene expression of control adipocytes and adipocytes differentiated with the excess of palmitic acid (16:0), stearic acid (18:0), and oleic acid (18:1n−9) obtained from SAT (**A**), VAT (**B**). The expression of lipid metabolism genes in adipocytes collected from SAT and VAT (**C**). The differential effect of fatty acid synthase (*FASN*) expression in cells collected from SAT and VAT differentiated with the excess of excess of palmitic acid (16:0), stearic acid (18:0) and oleic acid (18:1n−9) during adipogenesis **(D**); * *p* < 0.05, ** *p* < 0.001.

The influence of fatty acids on lipid metabolism was measured by the expression of lipid metabolism genes, that is one lipolysis (*LPL*—lipoprotein lipase) and three lipogenesis (*ACACA*—acetyl-CoA carboxylase α, *FASN*—fatty acid synthase, and *SCD1*—stearoyl-CoA desaturase 1). In cells collected from SAT, no changes in expression in experimental cells were detected compared to controls, except *SCD1* gene overexpression in cells treated with stearic acid (*p* = 0.0247, Figure 6A). However, in cells collected from VAT, numerous genes were overexpressed, including *LPL*, *FASN*, and *SCD1* in experimental cells compared to controls (Figure 6B). The *LPL* gene was overexpressed about 2.5 times more in cells cultured with palmitic (*p* = 0.0341) and stearic (*p* = 0.0314) acids compared with controls. Oleic acid stimulated the expression of *LPL* approximately two-fold more compared to controls (*p* = 0.0545). The expression of *FASN* was significantly overexpressed in cells cultured with stearic (*p* = 0.0086) and oleic (*p* = 0.0026) acids, whereas the *SCD1* gene was overexpressed in cells differentiated with the addition of palmitic (*p* = 0.0124) and stearic (*p* = 0.0008) acids.

Furthermore, we analyzed potential differences in lipid metabolism between cells collected from the two different fat depots. In particular, VAT adipocytes were characterized by significantly higher expression of the lipolytic gene (*LPL*, *p* = 0.0119), whereas lipogenesis genes were highly expressed in cells obtained from SAT; however, the difference was only nearly statistically significant (*p* = 0.0965, *p* = 0.0734, *p* = 0.0858 for *ACACA*, *FASN*, and *SCD-1*, respectively, Figure 6C). Furthermore, an additive effect of the examined fatty acids in *FASN* expression in cells collected from VAT compared to SAT was shown (16:0, *p* = 0.0459; 18:0, *p* = 0.0004; 18:1n−9, *p* < 0.0000, Figure 6D).

## 4. Discussion

In the present paper, we displayed the effect of fatty acids excess on adipocytes differentiation and the phenotype of mature adipocytes. All of the investigated fatty acids were shown to influence the phenotype of mature adipocytes via various effects, mainly through impairment of insulin signaling and dysregulation of adipokine secretion and expression of lipid metabolic genes.

We showed that all of the examined fatty acids, if supplied in excess amounts, may lead to obesity and may increase the risk of obesity-related disorders such as insulin resistance. First of all, we observed that an excess of oleic acid enhanced adipogenesis, as estimated based on the expression of transcription factors, total lipid accumulation, and changes in morphological structures of the cell during adipogenesis. We particularly observed changes regarding total lipid accumulation in mature adipocytes that was five times greater than in controls. Our results confirmed the adipogenic effect of oleic acid described previously by us and by others using the 3T3-L1 cell line and hen or bovine adipocytes [10,12,21,22]. Previously, it had been suggested that oleic acid aggravates adipogenesis via stimulation of *PPARG* [10,12], which is consistent with our results. We showed that oleic acid stimulated expression of almost all transcription factors. Indeed, oleic acid and other polyunsaturated fatty acids: Docosahexaenoic acid (DHA) and Eicosapentaenoic acid (EPA) were shown to act as natural ligands for PPARG, stimulating the expression of *PPARG, CEBPA*, and adiponectin [15,23]. On the other hand, inhibition of *PPARG* attenuates lipid formation and adipogenesis [24]. These results may explain the beneficial effect of oleic acid on overall glucose and lipid metabolism through increasing the plasticity of adipose tissues and promoting lipid accumulation and storage, thus reducing lipid accumulation in other tissue or organs, as well as overall lipotoxicity [25]. In addition to oleic acid, stearic acid was also shown to increase the expression of transcription factors as well as total lipid accumulation and the size of mature adipocytes. The effect may be indirect, as we showed increased expression of the *SCD-1* gene. Eventually, this could lead to the increased content of endogenous oleic acid; however, no evaluation of the lipid fraction was performed. The above finding can be supported by previous results, where an increased content of oleic acid was observed in 3T3-L1 cells with stable overexpression of the *SCD-1* gene [26]. On the other hand, it needs to be mentioned that both oleic and stearic acids impaired insulin-stimulated glucose uptake; thus, the beneficial effect of oleic acid should be more fully evaluated in terms of the insulin-signaling pathway in adipose tissue.

Considerable differences were observed in the process of adipogenesis between preadipocytes collected from the two main fat depots. The SAT-derived mature adipocytes accumulated a greater amount of total lipids than those collected from VAT. Similarly, the expression of early transcription factors was greater in adipocytes from SAT than from VAT, which is consistent with other reports [27]. Furthermore, as has been shown previously, adipocytes from subcutaneous fat are larger and accumulate more lipids than those from visceral fat in obese or overweight subjects [28], which is also consistent with our results. *PPARG* expression was much greater in adipocytes collected from VAT throughout the process of differentiation, especially in cells treated with oleate (18:1n−9) during adipogenesis.

In addition to their effect on the adipogenesis process, fatty acids impacted the metabolism of mature adipocytes. A constant excess of fatty acids during adipogenesis disrupted correct carbohydrate metabolism. Based on a gene expression study, insulin-signaling genes were not aborted; however, utilization of insulin-stimulated glucose by adipocytes differentiated, with an excess of all of the examined fatty acids discontinued. A similar effect was seen for all of the examined fatty acids, irrespective of the origin of the adipocytes. Treatment of cells with oleic acids reduced the rate of *SLC2A4* expression in SAT-derived adipocytes. On the other hand, we observed an increase in *SLC2A4* expression in VAT-derived adipocytes, which may be a compensatory effect of newly developed insulin resistance. We, and other researchers, have recently shown considerable reduction in the expression of *SLC2A4* in 3T3-L1 adipocytes [12] and bovine adipocytes [22] treated with oleic acid. However, further research needs to be conducted to evaluate the reason for the differential profile of *SLC2A4* expression between two fat depots influenced by oleic acid.

In SAT-derived adipocytes, the examined fatty acids displayed no divergences in the expression of enzymes regulating lipid metabolism, apart from overexpression of *SCD-1* in cells cultured with the addition of stearic acid, which may be due to increased access to the substrate for SCD-1. We observed a similar effect regarding 3T3-L1 adipocytes [12]. On the other hand, we showed dysregulation in the expression of lipid metabolism genes in VAT-derived adipocytes, including *LPL*, *FASN*, and *SCD1*, proving that VAT is metabolically more active than SAT. It had been concluded previously, based on results obtained in vivo and in vitro, that visceral fat was characterized by a higher level of lipolytic activity compared to subcutaneous fat [4]. This also explains the increased degree of lipid accumulation in SAT-derived adipocytes during adipogenesis compared to VAT-derived adipocytes, which could be explained in turn by the greater lipogenesis activity of SAT adipocytes and much lower levels of lipolysis activities in those cells. Contrary to SAT, VAT-derived adipocytes were characterized by a significantly higher level of lipolytic activity, which is consistent with other reports [4,29].

It is obvious that obesity is related to low-grade inflammation; accordingly, we were interested as to whether fatty acids were capable of influencing cytokine secretion. Generally, VAT is related to the development of inflammatory states mainly through the production of pro-inflammatory cytokines such as IL-6 [26]. Indeed, we showed that VAT-derived adipocytes expressed and secreted IL-6 at a higher rate than SAT-derived adipocytes; however, based on our study, fatty acids did not influence cytokine secretion. Our results contradict others that showed cytokine overexpression (*IL-6*, *TNFα* (Tumor Necrisis Factor α)) following palmitic acid treatment [30]. The increase in cytokine expression and secretion is likely visible at a later stage in the life of adipocytes. SAT and VAT also differ in terms of profiles of adipokine secretion. It had been previously reported that adipokines are expressed and secreted at a high level by SAT [31,32]. We, like others, showed significantly greater expression and secretion of leptin and adiponectin in SAT-derived adipocytes [33,34]. Furthermore, the influence of fatty acids was considerable, mainly on SAT. Generally, we reported that, in SAT-derived adipocytes, secretion of leptin was stimulated, and adiponectin secretion was inhibited by fatty acids. These results confirmed that insulin resistance developed in experimental cells, as adiponectin, irrespective of its other roles, was shown to increase insulin-stimulated glucose uptake [35]. We detected no changes in the expression or secretion rate of adiponectin in VAT-derived adipocytes, suggesting a minor role for VAT depots in the secretion of adiponectin. Indeed, the VAT-derived cells expressed and secreted less adiponectin than the adipocytes obtained from SAT.

In all cases, the above differences prove that SAT and VAT are metabolically different tissues; furthermore, dietary factors such as fatty acids have a different effect on both adipogenesis and the phenotype of mature adipocytes. Oleic acid was shown to stimulate adipogenesis of both SAT and VAT-derived MSCs and to increase total lipid accumulation. All of the examined fatty acids affected the phenotype and metabolism of mature adipocytes, albeit with a different effect on the origin of adipocytes.

## Figures and Tables

**Table 1:** Sequences of primers used in the study for gene expression analysis.

Gene Name	Sequence 5′→3′	Exon *	Length [bp]	R^2^ **
*CEBPB*	F: AGCACCACGACTTCCTCTC	1	54	0.99
	R: AGTTCTTGCCCCCGTAGTC			
*C/EBPδ*	F: ATGTACGACGACGAGAGCG	1	108	0.98
	R: GTTGAAGAGGTCGGCGAAG			
*C/EBPα*	F: GCCAAGAAGTCGGTGGACA	1	150	0.98
	R: GCGGTCATTGTCACTGGTC			
*PPARG*	F: TAATGCCATCAGGTTTGGGC	4/5	101	0.96
	R: GGTCAGCGGACTCTGGATT			
*INSR*	F: TTCGAGGAGGCAACAATCTG	4/5	93	0.95
	R: CGTAGGATCGGCGGATTTTT			
*PIK3R1*	F: TGCCTGCTCTGTAGTGGTG	14/15	70	0.98
	R: GCCATAGCCAGTTGCTGTTT			
*SLC2A4*	F: AGCAGCTCTCTGGCATCAAT	8/9	139	0.99
	R: ACCAACAACACCGAGACCAA			
*IL-6*	F: TCAATGAGGAGACTTGCCTG	2/3	114	0.98
	R: GCACAGCTCTGGCTTGTTC			
*IL-10*	F: GGACTTTAAGGGTTACCTGG	2/3	95	0.99
	R: CTGGGTCTTGGTTCTCAGC			
*ADIPOQ*	F: GGAGATCCAGGTCTTATTGG	1/2	184	0.99
	R: TGGGCATGTTGGGGATAGTA			
*LEP*	F: TTCACACACGCAGTCAGTCT	1/2	134	0.98
	R: GCATACTGGTGAGGATCTGT			
*LPL*	F: TCACTCTGCCTGAAGTTTCC	7/8	135	0.99
	R: TGCTCCACCAGTCTGACCA			
*ACACA*	F: CGTCCTCACCCAACCCAAA	47/48	105	0.98
	R: TCTACCAACCACCACAGTCT			
*FASN*	F: GGCATCAATGTCCTGCTGAA	4/5	107	0.99
	R: TACCCATTCCCCGCTGTGT			
*SCD-1*	F: CCAGAGGAGGTACTACAAAC	3/4	72	0.99
	R: AAATACCAGGGCACAAGCGT			
*Β-actin*	F: GAGAAGATGACCCAGATCA	2/3	72	0.99
	R: TAGCACAGCCTGGATAGCAA			

* the number of exons was estimated for the longest splicing variant except for *PPARG*, which was estimated for variant 2. ** the efficiency of the primer assessed based on a standard curve.
